# cN2–cN3 Penile Squamous Cell Carcinoma: Patient Selection, Treatment Delivery, and Real-World Challenges

**DOI:** 10.1590/S1677-5538.IBJU.2026.9911

**Published:** 2026-06-25

**Authors:** Pablo A. L. Mattos, Leonardo O. Reis

**Affiliations:** 1 Universidade Estadual de Campinas Faculdade de Ciências Médicas Campinas SP Brasil UroScience, Faculdade de Ciências Médicas, Universidade Estadual de Campinas - UNICAMP, Campinas, SP, Brasil; 2 Associação Piauiense de Combate ao Câncer Hospital São Marcos Teresina PI Brasil Associação Piauiense de Combate ao Câncer - Hospital São Marcos, Teresina, PI, Brasil; 3 Pontifícia Universidade Católica de Campinas Faculdade de Ciências da Vida Campinas SP Brasil ImunOncologia, Faculdade de Ciências da Vida, Pontifícia Universidade Católica de Campinas - PUC-Campinas, Campinas, SP, Brasil; 4 Instituto Nacional de Ciência, Tecnologia e Inovação em Câncer Urogenital INCT UroGen Campinas SP Brasil INCT UroGen, Instituto Nacional de Ciência, Tecnologia e Inovação em Câncer Urogenital, Campinas, SP, Brasil

## COMMENT

Regional lymph node metastasis remains the most important determinant of survival in penile squamous cell carcinoma (PSCC) (1). While outcomes for localized disease are generally favorable when appropriately managed, the development of clinically advanced nodal disease dramatically alters the natural history of PSCC. Patients presenting with cN2–cN3 involvement face high rates of recurrence, distant progression, and cancer-specific mortality, making this subgroup the greatest therapeutic challenge in contemporary penile cancer management and the leading cause of penile cancer-related death.

Historically, surgery represented the cornerstone of treatment for node-positive disease, based on the principle that complete surgical eradication of regional metastases could provide durable oncological control. However, clinical experience progressively demonstrated the limitations of surgery alone in patients with bulky, bilateral, fixed, or extranodal nodal metastases (2, 3). Even when technically feasible, surgical treatment was frequently followed by early recurrence and poor long-term survival in patients with advanced nodal disease, suggesting that locoregional control alone was insufficient for a subgroup of patients with biologically aggressive disease and a high likelihood of occult systemic dissemination.

These observations provided the rationale for introducing systemic therapy earlier in the treatment pathway. By addressing potential micro-metastatic disease, reducing tumor burden, and improving the likelihood of complete surgical resection, neoadjuvant chemotherapy (NAC) emerged as an attractive strategy for patients with locally advanced node-positive PSCC. The landmark phase II study by Pagliaro et al. demonstrated an objective response rate of approximately 50% with neoadjuvant TIP, establishing the foundation for contemporary treatment recommendations (4). The concept was further supported by experience with other squamous cell carcinomas, in which multimodal approaches had demonstrated advantages over local treatment alone in selected high-risk populations.

Over the last decade, NAC has progressively become the preferred treatment approach for patients with cN2–cN3 disease (1, 4). Contemporary evidence suggests that approximately half of patients achieve an objective response to neoadjuvant treatment, enabling potentially curative surgical consolidation (4, 5). In a recent international, multi-institutional study involving 209 patients with locally advanced, node-positive PSCC, the objective response rate was 57.2%, with a pathologic complete response rate of 24.8% in the lymph nodes, and 97% of patients completed the planned consolidative lymphadenectomy (5). As a result, contemporary international guidelines recommend platinum-based NAC followed by lymph node dissection in appropriately selected patients (1). Yet an important paradox remains despite its widespread acceptance, the evidence supporting NAC in PSCC is largely derived from small, non-randomized studies, leaving many clinically relevant questions unanswered (6).

The contemporary debate is no longer whether NAC should be considered for patients with cN2–cN3 PSCC. Current evidence and guideline recommendations have largely established its role in appropriately selected individuals (1, 4). Instead, the more relevant question is which patients are most likely to benefit from this strategy. Although treatment algorithms frequently group cN2 and cN3 disease into a single therapeutic category, current guidelines distinguish between patients with pelvic lymph node involvement or extensive inguinal disease, for whom NAC is preferred, and selected patients with bulky mobile bilateral inguinal disease, in whom NAC may be considered as an alternative to upfront surgery (1). Differences in nodal burden, laterality, fixation, extranodal extension, performance status, and comorbidities may substantially influence treatment tolerance, response, and ultimately long-term oncological outcomes.

Disease burden is likely one of the most important yet insufficiently explored determinants of NAC benefit (1). Patients with limited bilateral nodal involvement may differ substantially from those presenting with bulky, fixed, or clinically extranodal disease, despite being broadly classified within the same treatment category. This distinction may be particularly relevant in cN2 disease, where neoadjuvant chemotherapy remains an alternative rather than a preferred strategy, in contrast to patients with pelvic nodal involvement or extensive inguinal disease for whom NAC is generally favored (1). Although current evidence does not permit robust stratification of outcomes by specific patterns of nodal presentation, it is reasonable to assume that the biological behavior, probability of response, and feasibility of subsequent surgical consolidation are not uniform across this spectrum. Recognizing this heterogeneity may be essential to refining treatment decisions and designing future studies in locally advanced PSCC.

Patient-related factors deserve equal consideration when evaluating candidates for NAC. Beyond disease burden, the ability to tolerate and complete cisplatin-based treatment may substantially influence outcomes in patients with cN2–cN3 PSCC. Renal dysfunction, impaired performance status, neuropathy, and hearing loss may preclude the use of TIP, the preferred neoadjuvant regimen recommended by contemporary guidelines (1). For these patients, the decision to pursue alternative chemotherapy regimens or proceed directly to surgery remains poorly supported by high-quality evidence (7). Consequently, treatment selection should extend beyond anatomical staging to include a broader assessment of the patient's physiological reserve and capacity to successfully complete multimodal therapy.

An equally important and often underappreciated issue is treatment delivery. Discussions surrounding NAC in PSCC frequently focus on regimen selection and response rates, whereas considerably less attention has been paid to whether treatment is delivered as intended. Dose reductions, treatment delays, cycle omissions, and premature discontinuation may compromise the opportunity for timely surgical consolidation, particularly in patients with advanced nodal disease and limited physiological reserve. Consequently, the effectiveness of NAC may depend not only on the regimen prescribed but also on the ability to successfully deliver multimodal treatment according to plan.

Although rarely reported in penile cancer studies, treatment delivery metrics may represent important determinants of oncological outcomes. Delays in treatment initiation, reduced dose intensity, unplanned hospitalizations, and failure to complete the intended number of cycles have the potential to compromise both systemic disease control and the opportunity for timely surgical consolidation. In a recent international multi-institutional study, 97% of patients successfully completed the planned consolidative lymphadenectomy following NAC, suggesting that the effectiveness of multimodal treatment depends not only on treatment response but also on the successful delivery of the entire therapeutic strategy (5). This issue may be particularly relevant in PSCC, where the therapeutic window between potentially curable locoregional disease and irreversible progression can be narrow. Future investigations should move beyond conventional response endpoints and incorporate measures of treatment delivery to better understand the real-world effectiveness of NAC.

Importantly, NAC should not be viewed as a substitute for surgery. Rather, its primary purpose is to facilitate definitive surgical management by reducing tumor burden, increasing resectability, and potentially improving long-term disease control. The best oncological outcomes reported in the literature have consistently been observed among patients who respond to systemic therapy and subsequently undergo consolidative lymph node dissection (4, 5). In the largest contemporary real-world cohort, patients who achieved an objective response to NAC had a markedly longer median overall survival than non-responders (73.0 vs. 17.0 months, P < 0.01) (5). This observation reinforces the notion that surgery remains the cornerstone of curative-intent treatment, with NAC serving as an adjunct to optimize patient selection and enhance the effectiveness of locoregional therapy.

Response assessment after NAC represents another area of uncertainty in PSCC management. While patients achieving a marked clinical response are generally considered ideal candidates for consolidative surgery, the optimal approach for those with stable disease or incomplete responses remains less clear. In the absence of robust evidence, treatment decisions frequently rely on multidisciplinary discussion and individualized clinical judgment. Importantly, stable disease should not necessarily preclude surgical intervention, particularly when resection remains technically feasible and overt disease progression has been avoided. This pragmatic approach acknowledges both the biological heterogeneity of PSCC and the limitations of the current evidence base.

The future of NAC in PSCC will likely depend on moving beyond purely anatomical staging toward a more comprehensive understanding of both tumor biology and host factors. Emerging biomarkers, including HPV status, p16 expression, and tumor-infiltrating lymphocytes, may contribute to improved patient stratification and prediction of treatment response (8). At the same time, systemic factors such as sarcopenia and other body composition parameters have gained increasing attention as potential indicators of physiological reserve and treatment tolerance (9). Despite their potential clinical relevance, none of these factors have yet been incorporated into routine decision-making for patients undergoing NAC, highlighting an important opportunity for future investigation. Likewise, patient-reported outcomes and quality-of-life measures warrant greater consideration, particularly in a disease in which aggressive multimodal therapy may carry substantial physical and psychosocial consequences (10). Together, these dimensions may help define a more individualized approach to multimodal treatment in the years ahead.

Real-world implementation of NAC presents additional challenges that are often underrepresented in the available literature. In many regions where PSCC remains relatively prevalent, barriers such as delayed diagnosis, limited access to specialized multidisciplinary care, restricted availability of systemic therapy, and treatment interruptions may substantially influence outcomes. Under these circumstances, treatment delivery itself may become a major determinant of success, potentially influencing outcomes as much as the intrinsic efficacy of the selected regimen. This perspective highlights the importance of developing treatment strategies that are not only evidence-based but also adaptable to diverse healthcare environments.

Future research efforts should prioritize clinically meaningful questions that extend beyond conventional oncological endpoints. Despite an expanding literature, contemporary evidence remains largely derived from retrospective cohorts and small prospective studies, with no completed randomized trials available to guide practice (11). Collaborative prospective studies are needed to better define which patients derive the greatest benefit from NAC, establish standardized treatment delivery metrics, and clarify the optimal integration of systemic therapy with surgery. Equally important, the incorporation of biological markers, body composition analyses, and patient-reported outcomes may foster a more comprehensive understanding of treatment effectiveness (8, 9). Ultimately, progress in this field will depend not only on the development of novel therapies but also on generating evidence that reflects the complexity of real-world clinical practice and the priorities of patients living with PSCC.

Immunotherapy is emerging as a promising therapeutic strategy in advanced PSCC and may further reshape the management of patients with cN2–cN3 disease. Early-phase studies have demonstrated encouraging activity with both single-agent immune checkpoint inhibitors and chemoimmunotherapy combinations, although the available evidence remains limited by small sample sizes and the absence of randomized trials (12, 13). The HERCULES study reported an objective response rate of 39.4% with pembrolizumab combined with platinum-based chemotherapy in patients with advanced PSCC, supporting the rationale for further investigation of immune-based approaches (12). However, important questions remain regarding optimal patient selection, treatment sequencing, biomarker integration, and access to these therapies in regions where penile cancer is most prevalent. As with NAC, the successful incorporation of immunotherapy into clinical practice will depend not only on efficacy but also on the ability to deliver these treatments within real-world healthcare environments.

In conclusion, the management of cN2–cN3 PSCC has evolved considerably over the past decade, with multimodal treatment strategies increasingly shaping contemporary practice. However, important uncertainties remain regarding patient selection, treatment delivery, response assessment, and the incorporation of emerging biological and patient-centered factors into clinical decision-making. The expanding therapeutic landscape, including the emergence of immunotherapy-based approaches, further underscores the need for evidence to guide increasingly complex treatment decisions. Addressing these challenges will require collaborative efforts to generate data that are both scientifically rigorous and applicable to real-world practice. Ultimately, the next frontier in the management of cN2–cN3 PSCC lies not simply in developing new therapies, but in ensuring that the right patient receives the right multimodal strategy at the right time.

## Data Availability

All data generated or analysed during this study are included in the published article.

## References

[1] European Association of Urology (2024). EAU Guidelines on Penile Cancer.

[2] Ornellas AA, Seixas AL, Marota A, Wisnescky A, Campos F, de Moraes JR (1994). Surgical treatment of invasive squamous cell carcinoma of the penis: retrospective analysis of 350 cases. J Urol.

[3] Protzel C, Alcaraz A, Horenblas S, Pizzocaro G, Zlotta A, Hakenberg OW (2009). Lymphadenectomy in the surgical management of penile cancer. Eur Urol.

[4] Pagliaro LC, Williams DL, Daliani D, Williams MB, Osai W, Kincaid M (2010). Neoadjuvant paclitaxel, ifosfamide, and cisplatin chemotherapy for metastatic penile cancer: a phase II study. J Clin Oncol.

[5] Rose KM, Pham R, Zacharias NM, Ionescu F, Paravathaneni M, Marchetti KA (2024). Neoadjuvant Platinum-Based Chemotherapy and Lymphadenectomy for Penile Cancer: An International, Multi-Institutional, Real-World Study. J Natl Cancer Inst.

[6] Nicholson S, Hall E, Harland SJ, Chester JD, Pickering L, Barber J (2013). Phase II trial of docetaxel, cisplatin and 5-fluorouracil chemotherapy in locally advanced and metastatic penile cancer. Br J Cancer.

[7] Grudzińska M, Czajkowski M, Dolny M, Matuszewski M, Wierzbicki PM, Rybarczyk A (2025). Systemic Chemotherapy in Penile Squamous Cell Carcinoma: Mechanisms, Clinical Applications, and Evidence-Based Regimens. Cancers (Basel).

[8] Pekarek L, Ortega MA, Fraile-Martinez O, García-Montero C, Casanova C, Saez MA (2022). Clinical and Novel Biomarkers in Penile Carcinoma: A Prospective Review. J Pers Med.

[9] Hartmann V, Engelmann SU, Pickl C, Haas M, Kälble S, Goßler C (2024). Impact of sarcopenia and fat distribution on outcomes in penile cancer. Sci Rep.

[10] Torres Irizarry VM, Paster IC, Ogbuji V, Gomez DM, Mccormick K, Chipollini J (2024). Improving Quality of Life and Psychosocial Health for Penile Cancer Survivors: A Narrative Review. Cancers (Basel).

[11] Santucci J, Crisafi D, Sathianathen N, Eapen R, Bolton D, Murphy D (2026). Neoadjuvant Treatment for Penile Cancer: A Systematic Review of Contemporary Evidence. Cancers (Basel).

[12] Cotait Maluf F, Trindade K, Preto D, de Almeida Luz M, Medeiros Milhomem Beato P, Assed Bastos D (2025). Pembrolizumab Plus Platinum-Based Chemotherapy for Patients With Advanced Penile Cancer: The Nonrandomized HERCULES (LACOG 0218) Clinical Trial. JAMA Oncol.

[13] El Zarif T, Nassar AH, Pond GR, Zhuang TZ, Master V, Nazha B (2023). Safety and efficacy of immune checkpoint inhibitors in advanced penile cancer: report from the Global Society of Rare Genitourinary Tumors. J Natl Cancer Inst.

